# Morphofunctional Improvement of the Facial Nerve and Muscles with Repair Using Heterologous Fibrin Biopolymer and Photobiomodulation

**DOI:** 10.3390/ph16050653

**Published:** 2023-04-27

**Authors:** Cleuber Rodrigo de Souza Bueno, Maria Clara Cassola Tonin, Daniela Vieira Buchaim, Benedito Barraviera, Rui Seabra Ferreira Junior, Paulo Sérgio da Silva Santos, Carlos Henrique Bertoni Reis, Cláudio Maldonado Pastori, Eliana de Souza Bastos Mazuqueli Pereira, Dayane Maria Braz Nogueira, Marcelo Augusto Cini, Geraldo Marco Rosa Junior, Rogerio Leone Buchaim

**Affiliations:** 1 Department of Biological Sciences, Bauru School of Dentistry (FOB/USP), University of São Paulo, Bauru 17012-901, Brazil; cleuberbueno@usp.br (C.R.d.S.B.); mariaclaratonin@usp.br (M.C.C.T.); dr.carloshenriquereis@usp.br (C.H.B.R.); 2 Dentistry School, University Center of Adamantina (UNIFAI), Adamantina 17800-000, Brazil; claudiomaldonado@fai.com.br; 3 Medical School, University Center of Adamantina (UNIFAI), Adamantina 17800-000, Brazil; danibuchaim@alumni.usp.br; 4 Postgraduate Program in Structural and Functional Interactions in Rehabilitation, Postgraduate Department, University of Marilia (UNIMAR), Marília 17525-902, Brazil; elianabastos@unimar.br; 5 Center for the Study of Venoms and Venomous Animals (CEVAP), São Paulo State University (Universidade Estadual Paulista, UNESP), Botucatu 18610-307, Brazil; bbviera@gmail.com (B.B.); rui.seabra@unesp.br (R.S.F.J.); 6 Graduate Program in Tropical Diseases, Botucatu Medical School (FMB), São Paulo State University (UNESP—Universidade Estadual Paulista), Botucatu 18618-687, Brazil; 7 Department of Surgery, Stomatology, Pathology and Radiology, Bauru School of Dentistry, University of São Paulo, Bauru 17012-901, Brazil; paulosss@fob.usp.br; 8 UNIMAR Beneficent Hospital (HBU), University of Marilia (UNIMAR), Marília 17525-160, Brazil; 9 Department of Prosthodontics and Periodontics, Bauru School of Dentistry (FOB/USP), University of São Paulo, Bauru 17012-901, Brazil; dayanenogueira@usp.br; 10 Medical School, University of West Paulista (UNOESTE), Guarujá 11441-225, Brazil; marcelo.cini2@gmail.com; 11 Dentistry School, Faculty of the Midwest Paulista (FACOP), Piratininga 17499-010, Brazil; geraldomrjr@yahoo.com.br; 12 Graduate Program in Anatomy of Domestic and Wild Animals, Faculty of Veterinary Medicine and Animal Science, University of São Paulo (FMVZ/USP), São Paulo 05508-270, Brazil

**Keywords:** cranial nerve injuries, facial nerve, facial nerve diseases, low-level light therapy, fibrin tissue adhesive, biopolymers, transplantation, heterologous, facial muscles, facial expression

## Abstract

Peripheral nerve injuries impair the patient’s functional capacity, including those occurring in the facial nerve, which require effective medical treatment. Thus, we investigated the use of heterologous fibrin biopolymer (HFB) in the repair of the buccal branch of the facial nerve (BBFN) associated with photobiomodulation (PBM), using a low-level laser (LLLT), analyzing the effects on axons, muscles facials, and functional recovery. This experimental study used twenty-one rats randomly divided into three groups of seven animals, using the BBFN bilaterally (the left nerve was used for LLLT): Control group—normal and laser (CGn and CGl); Denervated group—normal and laser (DGn and DGl); Experimental Repair Group—normal and laser (ERGn and ERGl). The photobiomodulation protocol began in the immediate postoperative period and continued for 5 weeks with a weekly application. After 6 weeks of the experiment, the BBFN and the perioral muscles were collected. A significant difference (*p* < 0.05) was observed in nerve fiber diameter (7.10 ± 0.25 µm and 8.00 ± 0.36 µm, respectively) and axon diameter (3.31 ± 0.19 µm and 4.07 ± 0.27 µm, respectively) between ERGn and ERGl. In the area of muscle fibers, ERGl was similar to GC. In the functional analysis, the ERGn and the ERGI (4.38 ± 0.10) and the ERGI (4.56 ± 0.11) showed parameters of normality. We show that HFB and PBM had positive effects on the morphological and functional stimulation of the buccal branch of the facial nerve, being an alternative and favorable for the regeneration of severe injuries.

## 1. Introduction

The seventh pair of cranial nerves, the facial nerve, is responsible for the maintenance and dynamics of the muscles of facial expression [1,2], their injury can be caused by several factors such as facial trauma [3,4,5], tumors [6,7,8], iatrogenesis [2,9,10], viral infections [11,12] and metabolic diseases [13]. The impact of damage to this nerve includes facial aesthetic imbalance and loss of ophthalmic, nasal, and oral functions. Furthermore, there is a relevant psychological element involved, as it is directly related to the patient’s ability to socially interact and society’s negative perception of those with facial paralysis [14,15,16,17].

Peripheral nerve lesion levels define the extent of the prognosis for the patient. Pioneering, Seddon [18] developed a three-level classification involving the extent of damage to the axon and the connective tissue wrap of the nerve. The first degree, neuropraxia, is the milder degree and consists of a momentary functional decrease without direct nerve damage and support tissues, the next level is called axonotmesis, where we observe direct axon injury and local demyelination without loss of discontinuity of the axon structures. The most severe form that has an unfavorable prognosis is called neurotmesis, which involves the injury with total loss of axon discontinuity and connective tissue [19,20].

Given this, they would be important studies that propose to develop treatments or ways to accelerate axon regeneration to the target organ [21], especially neurotmesis, which necessarily surgical intervention is indicated [17,20,22]. The standard gold surgical treatment of neurotmesis without loss of tissue is end-to-end neurorrhaphy, with traditional suture coaptation [23,24,25,26]. However, sutures can trigger inflammatory processes, which can lead to neuroma formation and chronic neuropathic pain [27,28,29,30]. An alternative that has been tested is fibrin glue instead of traditional sutures with threads. However, they are expensive and contain fibrinogen and thrombin derived from human blood, which can transmit infectious and parasitic diseases [27,31,32].

Since 1990, the Center for the Study of Venoms and Venomous Animals (CEVAP/UNESP, Botucatu, Brazil) has been developing a new heterologous fibrin sealant. This is purified and extracted from snake venom (*Crotalus durissus terrificus*), and is biocompatible and biodegradable, hemostatic, adhesive, and does not produce adverse reactions [33]. Initially, fibrin sealant was used to glue nerves in preclinical studies and later used in clinical trials to treat chronic venous ulcers [34,35]. Due to its diverse biological properties, its production from animal products, and the possibility of use in different clinical situations, the name “heterologous fibrin sealant” was rethought, starting to be called “heterologous fibrin biopolymer” (HFB).

It proves to be a potentially risk-free method for the regenerative process, acting as a support and contributing to an axonal growth microenvironment in peripheral nerve injuries, obtaining promising results [36,37,38]. However, there is still no study with the coaptation of neurotmesis with the fibrin biopolymer in peripheral nerves analyzing the results in the innervated face muscles, so in this study, we perform the analysis of the cross-section area, which is a factor important for the functionality of the neuromuscular system [39,40,41].

However, in the search for faster and more effective morphological and functional recovery, the use of photobiomodulation (PBM) through the use of low-level laser therapy (LLLT) has been tested in peripheral nerves [10,42,43]. Effects such as increased mitotic activity and higher metabolic velocity, with a consequent increase in mitochondrial activity and transport of substances and oxygen, assist the activation of cell transcription factors, proliferation, survival and tissue repair, and nerve regeneration [44,45]. In addition, such benefits positively influence neuromuscular recovery when there are injured nerves, such as a decrease in the muscle degeneration process [46,47,48,49], but there is still no defined protocol in the literature for its use, and few studies with facial nerves.

Our group of researchers previously used a PBM protocol with the LLLT in the peripheral nerve defect repair process, obtaining promising results [37,38,45,50]. However, it is interesting to experiment with a new approach by modifying the frequency of treatment, aiming at better clinical adherence, patient convenience, and lower financial cost, increasing their use in clinical practice.

It can be hypothesized that HBF is effective for nerve repair and the PBM protocol used is capable of improving and accelerating the morphophysiological and functional recovery process. Therefore, the objective of this study was to investigate the repair of the buccal branch of the facial nerve using HFB as a means of coaptation of nerve stumps and LLLT with a new protocol with less frequency of applications, analyzing the effects on axonal and muscle regeneration and functional recovery.

## 2. Results

**Figure 1:**
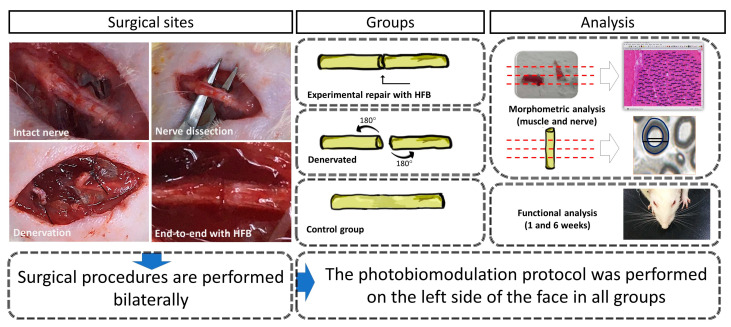
Surgical procedures and their treatments in the respective groups performed bilaterally. Morphological, morphometric, and functional evaluation.

The results were distributed into topics according to the analyzes carried out in the studied groups: control (Control Group, normal and laser—CGn and CGl), denervated without repairing surgical treatment (Denervated Group—normal and laser, DGn and DGl); experimental in which we performed the lesion and repair with HFB (Experimental Repair Group—normal and laser, ERGn and ERGl) after the surgical procedures and photobiomodulation protocol described in the experimental design (Figure 1). We also present qualitative and quantitative analyzes of the distal stump of the buccal branch of the facial nerve and facial muscles. Finally, we present the results of the functional analysis of the animals’ vibrissae.

### 2.1. Qualitative Nerve Analysis

**Figure 2:**
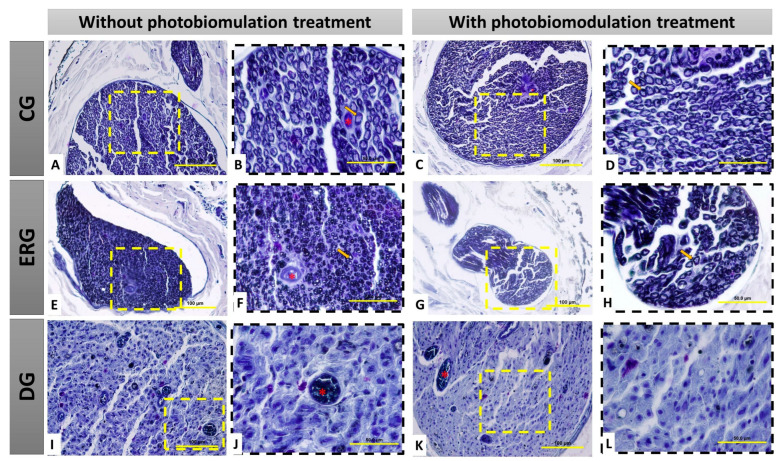
Histological view of the distal stump of the buccal branch of the facial nerve (BBFN) in cross-section, demonstrating the morphology in the different groups with or without photobiomodulation (PBM) treatment. In (**A**–**D**), seen at different magnifications of 40× (100 µm bar) and 100× (200 µm bar), there are myelin fibers with fascicular organization. In (**E**–**H**), seen at different magnifications (40 and 100×), it is observed that the groups with denervation and immediate repair with HFB also present myelin fibers, but in smaller size and less fascicular organization. In (**I**–**L**), seen at different magnifications of 40× (100 µm bar) and 100× (200 µm bar), the groups that underwent denervation and no surgical intervention was performed for repair, demonstrate severe morphological alterations of the distal stump of the nerve, resulting from this process, by observation the non-existence of myelin fibers, as well as a large invasion of scar tissue. CG = Control group, ERG= Experimental repair group, DG= Denervated group. Black arrow = myelin fiber; Asterisk (*) = blood vessel.

It was observed in the groups CGn and CGl organized and myelinic axonal fibers, fascicular histological architecture organized by the presence of conjunctive wraps, with perineurium and endoneurium delimiting each fascicle and axonal fiber (Figure 2A–D).

However, in the ERGn and ERGl groups, there was an invasion of dense connective tissue in connective envelopes, irregular axonal fibers, and visually smaller in relation to the CG, but regenerating reinnervated nerve fibers can be observed (Figure 2E–H). In addition, in the DGn and DGl histological slides, we observed the replacement of axonal fibers by dense connective tissue, highlighting the degenerated nervous tissue, demonstrating that the denervation was effective, and thus, in this group, it was not possible to perform the histomorphometric analysis (Figure 2I–L).

### 2.2. Histomorphometric Nerve Analysis

**Figure 3:**
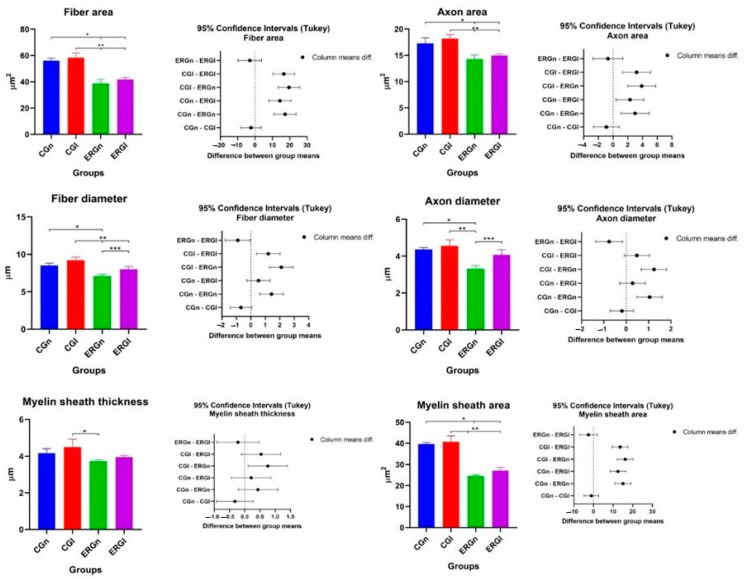
Histomorphometric results of the nerve (BBFN) of all studied groups demonstrated with mean and standard deviation column graph and standard deviation with the confidence intervals (Tukey). Asterisk (*, ** or ***) = significant difference between period/group (one-way ANOVA and Tukey, *p* < 0.05). CGn = Control group normal, CGl = Control group laser, ERGn = Experimental repair group normal, ERGl = Experimental repair group laser.

In the histomorphometry of the distal stump of the buccal branch of the facial nerve, it was observed, in the analysis of the areas of nerve fiber, axon, and myelin sheath, a significant difference between the control groups (GCn and CGl) and the experimental groups (ERGn and ERGl), and similarity between the experimental groups that were repaired with fibrin biopolymer, using or not the laser. Details of the mean and standard deviation values can be seen in Figure 3 and Table 1.

Table 1 shows the areas of the nerve fibers, axons, and myelin sheath, as well as the diameters of the nerve, axon, and myelin sheath. In the morphometric analysis of the nerve, in the DGn and DGl groups, it was not possible to carry out the measurements due to the absence of myelin fibers (Figure 2I–L). Therefore, groups DGn and DGl from Figure 3 and Table 1 were removed (values = zero).

Regarding the diameter, CGn showed a significant difference with ERGn in nerve and axon fiber diameter measurements. In CGl we observe a significant difference in nervous fiber with experimental groups (ERGn and ERGl) and a significant difference with ERGN in axon diameter and myelin sheath thickness with ERGn.

We also observe a significant difference in nerve fiber parameters and axons between experimental groups (ERGn and ERGl) with the highest averages for the group treated with photobiomodulation. More details of the values of average and default diameter deviation can be observed in Figure 3 and Table 1.

### 2.3. Qualitative Muscle Analysis

**Figure 4:**
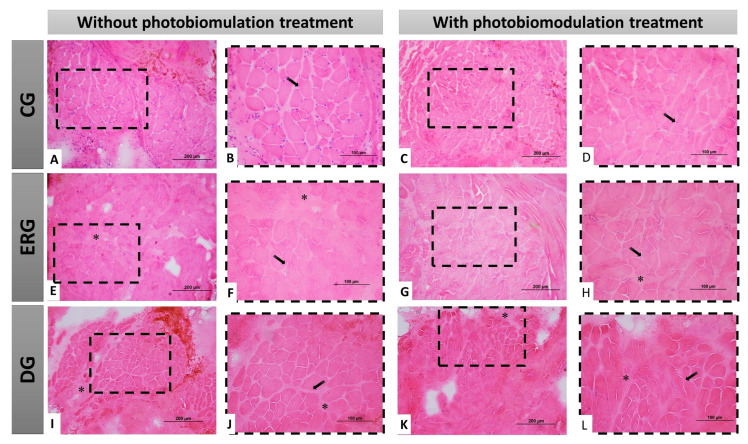
Histological view of the facial muscles in cross-section demonstrating the morphology of the different groups, with or without treatment with photobiomodulation. In (**A**–**D**), at different magnifications of 20× (200 µm bar) and 40× (100 µm bar), we observe polygonal muscle fibers, with peripheral nuclei and fascicular organization. In (**E**–**H**), at different magnifications (20 and 40×), the groups that underwent denervation and immediate repair with HFB, demonstrating a good histological pattern with few morphological changes. In (**I**–**L**), at different magnifications of 20× (200 µm bar) and 40× (100 µm bar), the groups that underwent denervation and no surgical intervention was performed for nerve repair, demonstrate some morphological alterations resulting from this situation, dimensional reduction of the muscle fibers and connective tissue invasion. CG = Control group, ERG = Experimental repair group, DG = Denervated group. Black arrow = muscle cell nucleus; Asterisk (*) intramuscular connective tissue.

In the control groups (CGn and CGl) were observed polygonal muscle fibers, with peripheral nuclei and organized histological architecture, visualized by the presence of conjunctive envelopments delimiting each fascicle and muscle fiber, highlighting the normal morphology of skeletal muscle tissue. In experimental groups (ERGn and ERGl), in some areas of the sample, we observe discreet invasion of connective tissue and muscle fibers with reduced cross-section area, permeated by larger section muscle fibers, however, continuous fascicular pattern and nuclei peripherals. Finally, in DGn and DGl groups, we observed the similarity of the qualitative pattern of experimental groups, however, with a greater number of muscle fibers with smaller area sections (Figure 4).

### 2.4. Histomorphometric Muscle Analysis

**Figure 5:**
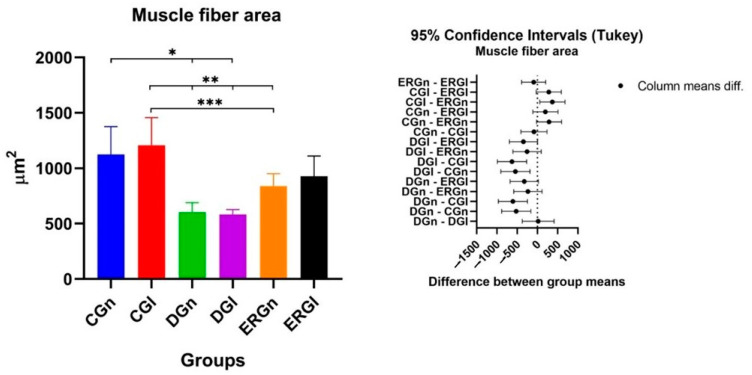
Mean and standard deviation of results of cross-section of muscle fibers and the difference between groups, demonstrated with mean and standard deviation column graph and standard deviation with the confidence intervals (Tukey). Asterisk (*, ** or ***) = significant difference between period/group (one-way ANOVA and Tukey, *p* < 0.05). CGn = Control group normal, CGl= Control group laser, DGn = Denervated group normal, DGl = Denervated group laser, ERGn = Experimental repair group normal, ERGl = Experimental repair group laser.

In Table 2, the results related to the facial muscle fiber area are indicated. We observed a statistical difference between the control groups (CGn and CGl) and the groups that were denervated without treatment (DGn and DGl). Experimental groups (ERGn and ERGl) were statistically similar. ERGN showed a statistical difference with CGl, and ERGl was similar to control groups. Figure 5 shows the graphs of the mean values and standard deviation with the confidence intervals (Tukey) of the morphometric analysis of the cross-sections of the muscle fibers in the study.

### 2.5. Functional Analysis of Whiskers Movements

Control groups (CGn and CGl) were used as a reference standard for the normality of whisker position and movement (score 5 for all animals), according to the parameters established by Faria et al. [51]. In the first week after surgery, we found similarities between experimental groups (ERGn and ERGl) and denervated groups (DGn and DGl), as well as significant differences with control groups.

**Figure 6:**
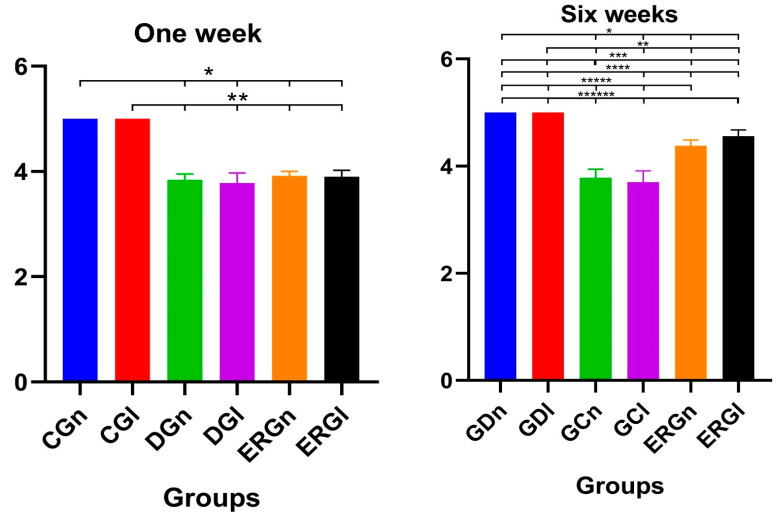
Functional results of vibrissae at 1 and 6 weeks postoperatively demonstrated with mean and standard deviation column graph. Asterisk (*, **, ***, ****, ***** or ******) = significant difference between period/group (one-way ANOVA and Tukey, *p* < 0.05). CGn = Control group normal, CGl = Control group laser, DGn = Denervated group normal, DGl = Denervated group laser, ERGn = Experimental repair group normal, ERGl = Experimental repair group laser.

The average score of ERGn was 3.90 ± 0.08 and the ERGl was 3.92 ± 0.12. In the sixth week after surgery, experimental groups showed similarities to each other and significant differences from denervated groups and controls. The average ERGn score was 4.38 ± 0.10 and the ERGl was 4.56 ± 0.11. The average values and standard deviation of the scores after functional analysis in 1 and 6 weeks after surgery are shown in Figure 6.

In a general context, the morphological and morphometric evaluations of the distal stump of the BBFN demonstrated that the denervation was effective, with axonal degeneration (DGn and DGl) and, in the groups reinnervated by neurorrhaphy with HFB (ERGn and ERGl), axonal sprouting occurred, but with fibers of disorganized orientation in relation to the controls (CGn and CGl). All measurements showed lower mean values for reinnervated groups compared to controls. In the facial muscles, negative repercussions of denervation were observed, with connective tissue invasion, but like the nerve, PBM improved the repair process qualitatively, quantitatively, and functionally.

## 3. Discussion

In the field of tissue bioengineering, which studies effective alternatives for the treatment of peripheral nerve injuries, we evaluated the regeneration of a lesion (neurotmesis) of the facial nerve using the fibrin biopolymer (HFB) and the association of photobiomodulation (PBM) with only a weekly application of LLLT for 5 weeks, performing morphological, morphometric (nervous and muscular) and functional evaluations. We observed that the use of HFB was an efficient means of coaptation for the differentiation of BBFN and that PBM promoted positive changes in the morphological aspects of the nerve. In the functional aspect, the groups with HFB repaired with or without LLLT at the end of 6 weeks, results were considered normal for the movement of the animals’ vibrissae.

HFB has been used for peripheral nerve repair, in order to replace or reduce the suture [52]. Studies comparing the use of fibrin sealants and sutures have shown less granulomatous inflammation, better axonal regeneration, and functional recovery in groups that used sealants for nerve coaptation [53,54,55]. With HFB we obtained the advantages of other sealants, but at a low cost, without adverse reactions or infections, due to the fact that it is produced with certified buffalo blood (*Bubalus bubalis*). Corroborating this fact, a study using HFB observed that its use reduced mechanical trauma to the nerve and reduced surgical time for peripheral nerve repairs, favoring the indication of this bioproduct [36].

The application of PBM in the regeneration of peripheral nerves has been reported in the literature with positive effects. Increased cellular metabolism, greater vascular budding, and collagen synthesis have been reported as indirect effects that aid in nerve regeneration. Added to this, increased Schwann cell proliferation, increased axonal growth velocity, and anti-inflammatory, analgesic, and anti-edema effects on the nerve have been described as positive effects directly related to nerve regeneration [45,56,57,58,59]. However, it is not yet clear which would be the most efficient protocols for each type of nerve (sensory, motor, or mixed), location (of the lesion and the effector organ), and classification of the lesion (neuropraxia, axonotmesis, or neurotmesis). In our study, it was thought of the use of a PBM protocol with LLLT using a single weekly application within 5 weeks after surgery, aiming at fewer clinical sessions, lower cost, and patient accessibility to treatment. The question would be whether therapy would maintain a positive photostimulant effect. Our group presents some previously published studies using an established parameter PBM protocol; however, using the application of therapy three times a week results in promising results in nerve regeneration of peripheral nerves [37,38,50].

Studies using 3 weekly applications for 4 weeks have been reported by other authors [59]. Lee et al. [60] demonstrated the increase in the diameter of axons using a wavelength of 604 nm. In our study, we also observed an increase in nerve fiber and axon diameter with 808 nm wavelength PBM. In another study performing axonotmesis, 980 nm PBM at three points along the nerve, three times a week for 5 weeks, had a beneficial effect on facial nerve regeneration, including the better functional capacity of the vibrissae and improvement in morphological nerve changes. Furthermore, there was a decrease and excessive suppression of apoptosis in Schwann cells induced by oxidative stress via activation of the PI3K/Akt signaling pathway [61].

However, PBM protocols with a single weekly dose are scarce in the literature. We aim to reduce the number of sessions, increasing the application time per point and, consequently, the energy per session. In the group that received PBM, after nerve repair, nerve, and axon diameters were similar to CG, suggesting that PBM with the once-weekly application protocol also accelerated nerve regeneration.

Regarding aspects of innervated muscles, after injury and during the period of nerve repair, neuromuscular changes occur, with consequent loss of trophic stimulus and, consequently, atrophy of muscle fibers and invasion of connective tissue [62,63,64]. Scarce scientific works have shown the effects on the target organ, being related to the success of neuromuscular regeneration [65], mainly in situations of neurotmesis. Thus, this study describes the effects of using HFB in the coaptation of neurotmesis, performing the histomorphometric analysis of the muscle innervated by BBFN. Thus, muscle preservation and reduction of the atrophic process in severe nerve injuries are challenges of regenerative medicine [66,67,68]. We observed that denervation led to a significant reduction in muscle fiber area in relation to the CG. However, the experimental groups showed less fiber atrophy, especially the group with PBM, which showed similarity in the area of muscle fibers with the GC, suggesting that the laser collaborated in reducing the post-repair atrophic process.

Therefore, regarding the functional analysis, BBFN is not the only one that provides motor innervation to rat vibrissae. When we only denervate this branch, we should not expect complete functional loss and total paralysis [69,70]. In the denervated group (DGn and DGl) there was loss of movement after 6 weeks, without total paralysis, probably due to having received innervation from another terminal branch of the facial nerve, such as the zygomatic. In ERGn and ERGl there was a normal movement score, according to the methodology of Faria et al. [51], not observing any difference between the groups with or without PBM.

The period of functional analysis the initial period to the postoperative (1 week) and 6 weeks postoperatively is described in studies in the literature [71,72,73,74]. Models such as the sciatic function index (SFI) and Basso, Beattie, and Bresnahan (BBB) are established in the literature for the sciatic nerve [71,72,75]. In a study that correlates these methods (SFI and BBB) for functional analysis, it is argued that the period of 6 weeks is adequate [72]. However, we must consider the different classifications of peripheral nerve injuries for the period of analysis, crush injuries (axoniotmesis), and neurotmesis without tissue loss. In a study that carried out the functional analysis of the facial nerve through the movement of the vibrissae using the total section of the nerve (neurotmesis) with and without tissue loss, a postoperative interval of four weeks was used. The authors noted that this was the time after surgery when the vibrissae began to demonstrate limited movement restitution in some animals. In the same study, they documented that during this period there was reinnervation of the motor plate in the levator labii superioris muscle, which is the largest muscle that innervates the vibrissae of animals, proving to be an interesting period for the analysis [76].

Another reason for choosing an initial and final period in our study was the fact that in 1 week after surgery, there is the presence of a post-trauma inflammatory process, making it possible to verify whether the use of LLLT in the initial phase could generate a functional gain. In the 6-week euthanasia period, the objective was to evaluate whether there was a gain in the functional speed of repair by comparing the groups that received or not LLLT. In addition, we used a shorter period of analysis of the results than in previous studies [38], which will allow a greater field of interpretation for future studies.

The denervated groups are important in this study because they allow us to observe the results of denervation in the facial muscles innervated by the buccal branch of the facial nerve. Added to this, we were able to compare the results of the surgical repair group with HFB both in the muscular and functional morphological scope with the vibrissae. The denervation technique used in the study, in which we performed a 180° rotation of the proximal and distal stump of the nerve and sutured it in the adjacent fascia and muscle, respectively, has already been proven to be effective in previous studies [40,75,77,78], not allowing spontaneous reinnervation. In this study, it was not possible to carry out the morphometric analysis of the distal stump of the buccal branch of the facial nerve in the DG groups, demonstrating the success of the technique, as there was a replacement of scar tissue and absence of myelin nerve fibers.

From future perspectives, the use of HFB associated with PBM in late repairs (not immediate to the injury) in neurotmesis can be investigated. An epidemiological study [79] revealed that 90% of the injuries caused were due to car accidents, an incidence that is increasing day by day compared to previous data [80]. Due to the morbidity of this type of trauma, nerve repair is often performed after days or weeks, so that there are stable conditions for the surgical procedure, which implies a longer period of convalescence. It is known that, in longer periods where the Schwann cells of the proximal stump of the injured nerve remain without contact with the axons of the distal stump after denervation, it is one of the main complicating factors, mainly in obtaining favorable results [62,81,82].

A possible limitation of this study was that we did not compare our previous protocol with three weekly sessions with the current protocol with fewer sessions; therefore, we cannot make any direct comparison between the protocols. In addition, in the present study, we used a device from another commercial brand, with greater power, which makes it difficult to directly associate the results. For the clinical translation of the association of HFB with PBM, it will be necessary to conclude phase III clinical studies of HFB, with relevant perspectives for use, as it has been shown to be a versatile and promising bioproduct in research in regenerative science.

## 4. Materials and Methods

The methodology used in this experimental protocol was based on previous studies by our group on peripheral nerve regeneration, causing an injury to the buccal branch of the facial nerve in rats [37,38,83]. In these studies, it was proven that HFB has the capacity to allow the repair of the lesion with results similar to the gold standard of end-to-end neurorrhaphy, which uses suture thread [84,85]. In an unprecedented way, in the present study, we evaluated a protocol with a smaller number of PBM sessions and also the effects on facial muscles, through morphofunctional analysis.

### 4.1. Experimental Design

This study was conducted in accordance with the Declaration of Helsinki, and approved by the Ethics Committee In Animal Use of the University of Marília (CEUA protocol code 033/2020 and date of approval 13 November 2020).

Twenty-one male Wistar rats (*Rattus norvegicus*) were used. The animals were 90 days old, weighing approximately 250–300 g at baseline. All animals were kept in appropriate boxes and received water and feed “ad libitum”, with no restrictions on movement, respecting the 12 h light/dark regime and an approximate temperature of 22 °C. Throughout the experimental period, signs and symptoms of stress and unusual behavior of the animals were observed. The study was carried out according to the ARRIVE protocol (animal research: report of in vivo experiments) and based on the principles of the NC3Rs (National Center for Replacement, Refinement, and Reduction of Research Animals).

The animals were randomly divided into groups (controls and experimental) of 7 animals, with no inclusion and exclusion criteria. The experiment was conducted with the buccal branch of the right and left facial nerves of all animals of the experiment. In all groups, it was performed on the left side the photobiomodulation therapy with the proposed protocol. We performed the euthanasia of all animals after 6 postoperative weeks.

The groups were named as follows: Control group—normal and laser (CGn and CGl): where the incision and dissection of the buccal branch of the facial nerve was performed bilaterally without injury to it; Denervated group—normal and laser (DGn and DGl): We performed neurotmesis bilaterally in these animals and did not perform any type of surgical repair; Experimental Group Repair—normal and laser (ERGn and ERGl): We performed neurotmesis and immediate repair with heterologous fibrin biopolymer bilaterally.

### 4.2. Heterologous Fibrin Biopolymer (HFB)

In the groups with neurotmesis repair, the heterologous fibrin biopolymer (HFB) was used. This material was provided by the Center for the Study of Venoms and Venomous Animals (CEVAP) of the São Paulo State University (UNESP), Botucatu, Brazil. HFB has 3 components that are defrosted and homogenized prior to application. In sequence, with the aid of a micropipette, the substances were applied for the coaptation of the stumps of the injured nerve. The first was the thrombin-like enzyme fraction (5 µL), the second contains calcium chloride diluent (5 µL), and the last was fibrinogen extracted from buffalo blood (10 µL). After application, 1 min was allowed for the polymerization of the biopolymer and then a slight traction of the nerves was performed to certify its adhesion.

The fibrin biopolymer components and application formula are in accordance with patent number BR 102014011432-7 issued on 6 July 2022 by the National Institute of Industrial Property of Brazil (INPI). This material underwent a phase I/II clinical trial [86] which proved its safety for therapeutic use in humans, standing out as a promising therapeutic potential.

### 4.3. Surgical Procedures

For all surgical procedures, the animals underwent general anesthesia with an intramuscular injection of tiletamine hydrochloride and zolazepam hydrochloride (10 mg/kg-Telazol^®^; Fort Dodge Laboratories, IA, USA). Trichotomy was performed with the aid of a hair trimmer (Philips^®^ Multigroom QG3250, São Paulo, Brazil) in the region of the bilateral face of the animals along the labial commissure to the tragus in order to obtain a smooth and hairless surface. Afterward, the animal was positioned in lateral decubitus in a surgical drip and antisepsis with 10% Polyvinyl Pyrrolidone Iodine PVPI (Povidine^®^ Antiseptic, Vic Pharma Ind e Comércio Ltd., São Paulo, Brazil)

#### 4.3.1. Denervation Surgery

A pre-auricular incision was made with blade 15 (Embramax^®^, São Paulo, Brazil) of approximately 3 cm with the aid of a surgical microscope (DF Vasconcelos^®^, São Paulo, Brazil). Subsequently, after division into planes, recognition, release, and sectioning of the buccal branch of the facial nerve (neurotmesis) were carried out in its central portion (point from the center of the line of the tragus to the labial commissure in lateral norm).

In DGn and DGl, in order to avoid spontaneous regeneration, the proximal stump was manipulated 180° degrees and sutured to the adjacent muscle fascia; the distal stump was manipulated 180° degrees and sutured to the adjacent musculature, both with 6-0 nylon thread [40,51,75]. The skin suture was performed with simple stitches using 4-0 Ethicon^®^ nylon thread (Johnson & Johnson Ind e Comércio Ltd., São Paulo, Brazil).

#### 4.3.2. Surgical Protocol for the Experimental Groups with Repair of the Buccal Branch of the Facial Nerve

After following the same steps described in 4.3.1 (Denervation surgery), the anatomical approximation of the sectioned nerve stumps was performed, without tension, and coaptation with fibrin biopolymer (see Figure 1) [37,51]. After restoring the neural continuity, the skin was sutured with simple stitches using 4-0 Ethicon^®^ nylon thread (Johnson & Johnson Ind e Comércio Ltd., São Paulo, Brazil).

#### 4.3.3. Post-Surgical Care

Immediately after the surgical procedures, the animals received a single dose of the antibiotic Flotril^®^ 2.5% (Schering-Plough, Rio de Janeiro, Brazil), at a dosage of 0.2 mL/kg and analgesic Dipyrone Analgex V^®^ (Agener União, São Paulo, Brazil) at a dose of 0.06 mL/kg in intramuscular applications. The application of the analgesic was maintained for 3 days, in addition to continuation with the analgesic Paracetamol^®^ (Generic medication, Medley, São Paulo, Brazil) at a dose of 200 mg/Kg, 6 drops/animal dissolved in the water available in the drinker so far of euthanasia.

### 4.4. Photobiomodulation Protocol (PBM)

**Figure 7:**
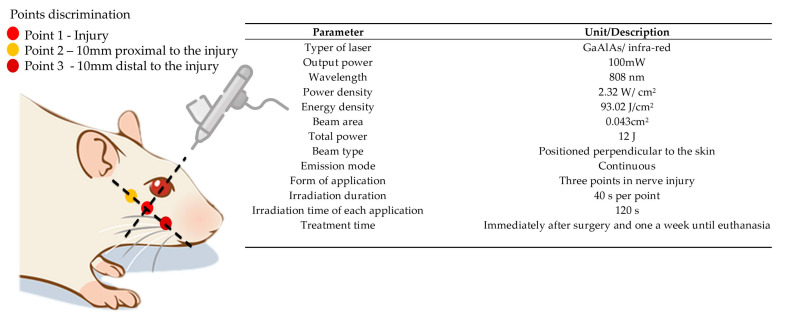
PBM protocol (Therapy XT DMC^®^ equipment, São Carlos, Brazil).

The treatment began in the immediate postoperative period and continued for 5 weeks with a weekly application, always occurring on the same day of the week throughout the protocol. The animals were manually immobilized (delicate restraint) and sedation was unnecessary during the application of photobiomodulation. All study groups received the PBM protocol in the buccal branch of the facial nerve on the left side using the protocol using the low-level laser of gallium aluminum arsenide (GaAlAs)—Therapy XT DMC^®^ (São Carlos, Brazil), with three application points along the path of the injured nerve, each point received an energy dose of 4 J, corresponding to 40 s per point, more details of the parameters are shown in Figure 7. Prior to the applications, the device was calibrated and tested to certify the dose.

### 4.5. Functional Analysis

Observations of the animals’ vibrissae movements were performed between 1 and 6 weeks postoperatively. The animals were placed in a box with a black background and the vibrissae were observed with spontaneous movements and when stimulated by the researcher (clap your hands 3 to 4 times), with the aim of triggering movements. The evaluator did not know which group was being evaluated (blind evaluation). After the observations, scores were assigned, following the methodology of Faria et al. [51]. During the observation, photographs of the animals were also taken.

### 4.6. Sample Collection and Euthanasia

After six weeks, the animals were anesthetized and the buccal branch of the facial nerve was carefully dissected and 10 mm of the distal stump from the neurotmesis site was collected in the experimental and denervated groups, as well as the intact nerve in the control group, under the magnified view of 16× of the surgical microscope (DF Vasconcelos^®^, São Paulo, Brazil). Next, the muscles of facial expression in the perioral region of all groups were dissected and carefully removed. Euthanasia was performed in a silent environment and away from the other animals, using an anesthetic overdose (triple dose—240 mg/kg of tiletamine hydrochloride + 30 mg/kg of zolazepam hydrochloride).

### 4.7. Histological Processing of Nerve and Muscle

The samples were fixed in a 10% buffered formaldehyde solution for 24 h and the historesin protocol was performed (Leica Mycrosistems^®^, Wetzlar, Germany) [38]. Sections were performed using a semiautomatic microtome (Model RM2245, Leica Microsystems^®^, Wetzlar, Germany) with a thickness of 5 µm. The slides were stained with Osmium Tetroxide and counterstained with 1% Toluidine Blue in distilled water. The sections were analyzed under an optical microscope.

Muscle samples were reduced to cylindrical fragments preserving the muscle belly, wrapped in surgical talc, immersed in liquid nitrogen, and included with an adhesive (Optimal Critical Temperature Tissue-Tek^®^ (O.C.T., Sakura Finetek, Torrance, CA, USA)). Then, samples were kept in a freezer at −80 °C until the ten micrometer-thick histological sections were obtained in a cryostat (Model CM 1850, Leica Microsystems^®^, Wetzlar, Germany) at −20 °C, which were stained with hematoxylin and eosin (HE).

### 4.8. Histological Analysis of Nerve and Muscle

The morphometry of the distal region of the buccal branch of the facial nerve was performed with the measurement of 220 fibers of the nerve and muscle of all samples of each group using a microcomputer coupled to a photomicroscope (Olympus^®^ BX50, Tokyo, Japan) and using a software of image capture and analysis (Image Pro-Plus^®^ 6.2—Media Cybernetics, Bethesda, MD, USA). The morphometric variables studied in nerves were: the area of nerve fibers, the area of axons, the minimum diameter of nerve fibers, the minimum diameter of axons, myelin sheath area, and myelin sheath thickness [37,38,50,83], and in muscles the cross-sectional area of muscle fibers was measured [40,75,87].

### 4.9. Statistical Analysis

Data were organized into spreadsheets and tables in Excel format (Microsoft Office Excel^®^, Redmond, WA, USA) with means and standard deviation, which were subsequently submitted to statistical tests. We used the two-way variance test (ANOVA) and then Tukey’s test for multiple comparisons between means. Statistical analyses and graphs were performed using the Graph Pad Prism version 8.0 program (GraphPad^®^ Software, La Jolla, CA, USA). The level of statistical significance was set at *p* < 0.05 for all analyses.

## 5. Conclusions

In order to optimize the morphofunctional recovery of peripheral nerve injuries, we investigated the use of heterologous fibrin biopolymer (HFB) in the repair of the buccal branch of the facial nerve (BBFN) associated with photobiomodulation (PBM). Here we show that the use of this bioproduct (HFB) to reconnect nerves was effective in allowing axonal growth in the stump distal to the lesion and minimizing the effects on innervated muscles. There was an improvement in the functionality of the vibrissae, and the biostimulatory effects of PBM showed efficacy for nerve and muscle repair. Therefore, this study demonstrates a regenerative technique with the association of a versatile bioproduct and PBM with a single weekly application of LLLT, proving its translational potential for clinical studies in tissue bioengineering.

## Figures and Tables

**Table 1:** Mean and standard deviation of histomorphometric analysis of the distal stump of the buccal branch of the facial nerve.

Groups	Fiber Area (µm^2^)	Axon Area (µm^2^)	Fiber Diameter (µm)	Axon Diameter (µm)	Myelin Sheath Area (µm^2^)	Myelin Sheath Thickness (µm)
CGn	56.11 ± 1.93	17.27 ± 1.07	8.53 ± 0.28	4.36 ± 0,10	39.65 ± 0,70	4.16 ± 0.24
CGl	58.37 ± 3.66	18.17 ± 0.87	9.21 ± 0.43	4.55 ± 0.33	40,74 ± 2.81	4.49 ± 0.42
ERGn	38.77 ± 2.96	14.28 ± 0.77	7.10 ± 0.25	3.31 ± 0.19	24.57 ± 0.57	3.73 ± 0.08
ERGl	41.77 ± 1.50	14.97 ± 0.28	8.00 ± 0.36	4.07 ± 0.27	27.13 ± 1.46	3.95 ± 0.09

CGn = Control group normal, CGl = Control group laser, ERGn = Experimental repair group normal, ERGl = Experimental repair group laser.

**Table 2:** Median and standard deviation of histomorphometric analysis of the area of facial muscle fibers.

Groups	Fiber Muscle Area (µm^2^)
CGn	1126.00 ± 250.50
CGl	1208.00 ± 249.60
DGn	603.60 ± 85.54
DGl	583.00 ± 44.36
ERGn	837.30 ± 113.80
ERGl	926.90 ± 183.60

CGn = Control group normal, CGl = Control group laser, DGn = Denervated group normal, DGl = Denervated group laser, ERGn = Experimental repair group normal, ERGl = Experimental repair group laser.

## Data Availability

The data presented in this study are available on request from the corresponding author.

## References

[1] Yang S.H., Park H., Yoo D.S., Joo W., Rhoton A. (2021). Microsurgical Anatomy of the Facial Nerve. Clin. Anat..

[2] Stuzin J.M., Rohrich R.J. (2020). Facial Nerve Danger Zones. Plast. Reconstr. Surg..

[3] Lam A.Q., Tran Phan Chung T., Tran Viet L., Do Quang H., Tran Van D., Fox A.J. (2022). The Anatomic Landmark Approach to Extratemporal Facial Nerve Repair in Facial Trauma. Cureus.

[4] Maxwell A.K., Lemoine J.C., Kahane J.B., Gary C.C. (2022). Management of the Facial Nerve Following Temporal Bone Ballistic Injury. Laryngoscope Investig. Otolaryngol..

[5] Markiewicz M.R., Callahan N., Miloro M. (2021). Management of Traumatic Trigeminal and Facial Nerve Injuries. Oral Maxillofac. Surg. Clin. North. Am..

[6] Cho Y.S., Choi J.E., Lim J.H., Cho Y.-S. (2022). Management of Facial Nerve Schwannoma: When Is the Timing for Surgery. Eur. Arch. Oto-Rhino-Laryngol..

[7] Guntinas-Lichius O., Silver C.E., Thielker J., Bernal-Sprekelsen M., Bradford C.R., de Bree R., Kowalski L.P., Olsen K.D., Quer M., Rinaldo A. (2018). Management of the Facial Nerve in Parotid Cancer: Preservation or Resection and Reconstruction. Eur. Arch. Oto-Rhino-Laryngol..

[8] Psillas G., Constantinidis J. (2023). Facial Palsy Secondary to Cholesteatoma: A Case-Series of 14 Patients. Audiol. Res..

[9] Zourntou S.-E., Makridis K.G., Tsougos C.-I., Skoulakis C., Vlychou M., Vassiou A. (2021). Facial Nerve: A Review of the Anatomical, Surgical Landmarks and Its Iatrogenic Injuries. Injury.

[10] Green J.D., Shelton C., Brackmann D.E. (1994). Iatrogenic facial nerve injury during otologic surgery. Laryngoscope.

[11] Spencer C.R., Irving R.M. (2016). Causes and Management of Facial Nerve Palsy. Br. J. Hosp. Med..

[12] Kim Y.-H., Kim J.-E., Yoon B.-A., Kim J.-K., Bae J.-S. (2022). Bilateral Facial Weakness with Distal Paresthesia Following COVID-19 Vaccination: A Scoping Review for an Atypical Variant of Guillain–Barré Syndrome. Brain Sci..

[13] Jung S.Y., Jung J., Byun J.Y., Park M.S., Kim S.H., Yeo S.G. (2018). The Effect of Metabolic Syndrome on Bell’s Palsy Recovery Rate. Acta Otolaryngol..

[14] Parsa K.M., Hancock M., Nguy P.L., Donalek H.M., Wang H., Barth J., Reilly M.J. (2020). Association of Facial Paralysis with Perceptions of Personality and Physical Traits. JAMA Netw. Open.

[15] Okuma H., Nagano R., Takagi S. (2012). Hemiplegic Peripheral Neuropathy Accompanied with Multiple Cranial Nerve Palsy. Clin. Pract..

[16] Li M.K.K., Niles N., Gore S., Ebrahimi A., McGuinness J., Clark J.R. (2016). Social Perception of Morbidity in Facial Nerve Paralysis. Head Neck.

[17] Fliss E., Yanko R., Zaretski A., Tulchinsky R., Arad E., Kedar D.J., Fliss D.M., Gur E. (2022). Facial Nerve Repair Following Acute Nerve Injury. Arch. Plast. Surg..

[18] Seddon H.J. (1943). Three Types of Nerve Injury. Brain.

[19] Menorca R.M.G., Fussell T.S., Elfar J.C. (2013). Nerve Physiology. Hand Clin..

[20] Krauss E.M., Weber R.V., Mackinnon S.E. (2022). Nerve Injury, Repair, and Reconstruction. Plastic Surgery—Principles and Practice.

[21] Riccio M., Marchesini A., Pugliese P., Francesco F. (2019). Nerve Repair and Regeneration: Biological Tubulization Limits and Future Perspectives. J. Cell Physiol..

[22] Grinsell D., Keating C.P. (2014). Peripheral Nerve Reconstruction after Injury: A Review of Clinical and Experimental Therapies. Biomed. Res. Int..

[23] Panagopoulos G.N., Megaloikonomos P.D., Mavrogenis A.F. (2017). The Present and Future for Peripheral Nerve Regeneration. Orthopedics.

[24] Lundborg G. (2000). A 25-Year Perspective of Peripheral Nerve Surgery: Evolving Neuroscientific Concepts and Clinical Significance. J. Hand Surg. Am..

[25] Rönkkö H., Göransson H., Taskinen H.-S., Paavilainen P., Vahlberg T., Röyttä M. (2016). Comparison of Peripheral Nerve Regeneration with Side-to-Side, End-to-Side, and End-to-End Repairs. Plast. Reconstr. Surg. Glob. Open.

[26] Battiston B., Artiaco S., Conforti L.G., Vasario G., Tos P. (2009). End-to-Side Nerve Suture in Traumatic Injuries of Brachial Plexus: Review of the Literature and Personal Case Series. J. Hand Surg. Eur. Vol..

[27] Chimutengwende-Gordon M., Khan W. (2012). Recent Advances and Developments in Neural Repair and Regeneration for Hand Surgery. Open Orthop. J..

[28] Wang W., Degrugillier L., Tremp M., Prautsch K., Sottaz L., Schaefer D.J., Madduri S., Kalbermatten D. (2018). Nerve Repair With Fibrin Nerve Conduit and Modified Suture Placement. Anat. Rec..

[29] Chow N., Miears H., Cox C., MacKay B. (2021). Fibrin Glue and Its Alternatives in Peripheral Nerve Repair. Ann. Plast. Surg..

[30] Bora F.W., Pleasure D.E., Didizian N.A. (1976). A Study of Nerve Regeneration and Neuroma Formation after Nerve Suture by Various Techniques. J. Hand Surg. Am..

[31] Martins R.S., Siqueira M.G., da Silva C.F., Plese J.P.P. (2005). Overall Assessment of Regeneration in Peripheral Nerve Lesion Repair Using Fibrin Glue, Suture, or a Combination of the 2 Techniques in a Rat Model. Which Is the Ideal Choice?. Surg. Neurol..

[32] Choi B.-H., Han S.-G., Kim S.-H., Zhu S.-J., Huh J.-Y., Jung J.-H., Lee S.-H., Kim B.-Y. (2005). Autologous Fibrin Glue in Peripheral Nerve Regeneration in Vivo. Microsurgery.

[33] Ferreira R.S., de Barros L.C., Abbade L.P.F., Barraviera S.R.C.S., Silvares M.R.C., de Pontes L.G., dos Santos L.D., Barraviera B. (2017). Heterologous Fibrin Sealant Derived from Snake Venom: From Bench to Bedside—An Overview. J. Venom. Anim. Toxins Incl. Trop. Dis..

[34] Barros L.C., Ferreira R.S., Barraviera S.R.C.S., Stolf H.O., Thomazini-Santos I.A., Mendes-Giannini M.J.S., Toscano E., Barraviera B. (2009). A New Fibrin Sealant From *Crotalus Durissus Terrificus* Venom: Applications in Medicine. J. Toxicol. Environ. Health B Crit. Rev..

[35] Gatti M., Vieira L., Barraviera B., Barraviera S. (2011). Treatment of Venous Ulcers with Fibrin Sealant Derived from Snake Venom. J. Venom. Anim. Toxins Incl. Trop. Dis..

[36] Leite A.P.S., Pinto C.G., Tibúrcio F.C., Sartori A.A., de Castro Rodrigues A., Barraviera B., Ferreira R.S., Filadelpho A.L., Matheus S.M.M. (2019). Heterologous Fibrin Sealant Potentiates Axonal Regeneration after Peripheral Nerve Injury with Reduction in the Number of Suture Points. Injury.

[37] Buchaim D.V., Andreo J.C., Ferreira Junior R.S., Barraviera B., de Rodrigues A.C., de Macedo M.C., Rosa Junior G.M., Shinohara A.L., Santos German I.J., Pomini K.T. (2017). Efficacy of Laser Photobiomodulation on Morphological and Functional Repair of the Facial Nerve. Photomed. Laser Surg..

[38] De Rosso M.P.O., Rosa Júnior G.M., Buchaim D.V., German I.J.S., Pomini K.T., de Souza R.G., Pereira M., Favaretto Júnior I.A., de Souza Bueno C.R., de Oliveira Gonçalves J.B. (2017). Stimulation of Morphofunctional Repair of the Facial Nerve with Photobiomodulation, Using the End-to-Side Technique or a New Heterologous Fibrin Sealant. J. Photochem. Photobiol. B.

[39] Lee J.I., Gurjar A.A., Talukder M.A.H., Rodenhouse A., Manto K., O’Brien M., Govindappa P.K., Elfar J.C. (2020). A Novel Nerve Transection and Repair Method in Mice: Histomorphometric Analysis of Nerves, Blood Vessels, and Muscles with Functional Recovery. Sci. Rep..

[40] de Bueno C.R.S., Pereira M., Favaretto Junior I.A., Bortoluci C.H.F., dos Santos T.C.P., Dias D.V., Daré L.R., Rosa Junior G.M. (2017). Electrical Stimulation Attenuates Morphological Alterations and Prevents Atrophy of the Denervated Cranial Tibial Muscle. Einstein.

[41] Bertin J.S.F., Marques M.J., Macedo A.B., de Carvalho S.C., Neto H.S. (2022). Effect of Photobiomodulation on Denervation-Induced Skeletal Muscle Atrophy and Autophagy: A Study in Mice. J. Manip. Physiol. Ther..

[42] Pasquale C., Utyuzh A., Mikhailova M.V., Colombo E., Amaroli A. (2021). Recovery from Idiopathic Facial Paralysis (Bell’s Palsy) Using Photobiomodulation in Patients Non-Responsive to Standard Treatment: A Case Series Study. Photonics.

[43] Hakimiha N., Rokn A.R., Younespour S., Moslemi N. (2020). Photobiomodulation Therapy for the Management of Patients With Inferior Alveolar Neurosensory Disturbance Associated With Oral Surgical Procedures: An Interventional Case Series Study. J. Lasers Med. Sci..

[44] Dias F.J., Fazan V.P.S., Cury D.P., de Almeida S.R.Y., Borie E., Fuentes R., Coutinho-Netto J., Watanabe I. (2019). Growth Factors Expression and Ultrastructural Morphology after Application of Low-Level Laser and Natural Latex Protein on a Sciatic Nerve Crush-Type Injury. PLoS ONE.

[45] Rosso M., Buchaim D., Kawano N., Furlanette G., Pomini K., Buchaim R. (2018). Photobiomodulation Therapy (PBMT) in Peripheral Nerve Regeneration: A Systematic Review. Bioengineering.

[46] Gigo-Benato D., Russo T.L., Tanaka E.H., Assis L., Salvini T.F., Parizotto N.A. (2010). Effects of 660 and 780 Nm Low-Level Laser Therapy on Neuromuscular Recovery after Crush Injury in Rat Sciatic Nerve. Lasers Surg. Med..

[47] Andraus R.A.C., Maia L.P., de Souza Lino A.D., Fernandes K.B.P., de Matos Gomes M.V., de Jesus Guirro R.R., Barbieri C.H. (2017). LLLT Actives MMP-2 and Increases Muscle Mechanical Resistance after Nerve Sciatic Rat Regeneration. Lasers Med. Sci..

[48] Mandelbaum-Livnat M.M., Almog M., Nissan M., Loeb E., Shapira Y., Rochkind S. (2016). Photobiomodulation Triple Treatment in Peripheral Nerve Injury: Nerve and Muscle Response. Photomed. Laser Surg..

[49] Rochkind S., Geuna S., Shainberg A. (2013). Phototherapy and nerve injury: Focus on muscle response. Int. Rev. Neurobiol..

[50] Buchaim R.L., Andreo J.C., Barraviera B., Ferreira Junior R.S., Buchaim D.V., Rosa Junior G.M., de Oliveira A.L.R., de Castro Rodrigues A. (2015). Effect of Low-Level Laser Therapy (LLLT) on Peripheral Nerve Regeneration Using Fibrin Glue Derived from Snake Venom. Injury.

[51] de Faria S.D., Testa J.R.G., Borin A., Toledo R.N. (2006). Standardization of Techniques Used in Facial Nerve Section and Facial Movement Evaluation in Rats. Braz. J. Otorhinolaryngol..

[52] Koopman J.E., Duraku L.S., de Jong T., de Vries R.B.M., Michiel Zuidam J., Hundepool C.A. (2022). A Systematic Review and Meta-Analysis on the Use of Fibrin Glue in Peripheral Nerve Repair: Can We Just Glue It?. J. Plast. Reconstr. Aesthet. Surg..

[53] Sameem M., Wood T.J., Bain J.R. (2011). A Systematic Review on the Use of Fibrin Glue for Peripheral Nerve Repair. Plast. Reconstr. Surg..

[54] Palazzi S., Vila-Torres J., Lorenzo J. (1995). Fibrin Glue Is A Sealant and Not a Nerve Barrier. J. Reconstr. Microsurg..

[55] Rafijah G., Bowen A.J., Dolores C., Vitali R., Mozaffar T., Gupta R. (2013). The Effects of Adjuvant Fibrin Sealant on the Surgical Repair of Segmental Nerve Defects in an Animal Model. J. Hand Surg. Am..

[56] Modrak M., Talukder M.A.H., Gurgenashvili K., Noble M., Elfar J.C. (2020). Peripheral Nerve Injury and Myelination: Potential Therapeutic Strategies. J. Neurosci. Res..

[57] della Santa G.M.L., Ferreira M.C., Machado T.P.G., Oliveira M.X., Santos A.P. (2021). Effects of Photobiomodulation Therapy (LED 630 Nm) on Muscle and Nerve Histomorphometry after Axonotmesis. Photochem. Photobiol..

[58] Faroni A., Mobasseri S.A., Kingham P.J., Reid A.J. (2015). Peripheral Nerve Regeneration: Experimental Strategies and Future Perspectives. Adv. Drug. Deliv. Rev..

[59] Andreo L., Soldera C.B., Ribeiro B.G., de Matos P.R.V., Bussadori S.K., Fernandes K.P.S., Mesquita-Ferrari R.A. (2017). Effects of Photobiomodulation on Experimental Models of Peripheral Nerve Injury. Lasers Med. Sci..

[60] Lee J., Carpena N.T., Kim S., Lee M.Y., Jung J.Y., Choi J.E. (2021). Photobiomodulation at a Wavelength of 633 Nm Leads to Faster Functional Recovery than 804 Nm after Facial Nerve Injury. J. Biophotonics.

[61] Li B., Wang X. (2022). Photobiomodulation Enhances Facial Nerve Regeneration via Activation of PI3K/Akt Signaling Pathway–Mediated Antioxidant Response. Lasers Med. Sci..

[62] Gordon T. (2020). Peripheral Nerve Regeneration and Muscle Reinnervation. Int. J. Mol. Sci..

[63] Geuna S., Raimondo S., Ronchi G., di Scipio F., Tos P., Czaja K., Fornaro M. (2009). Chapter 3 Histology of the Peripheral Nerve and Changes Occurring During Nerve Regeneration. Int. Rev. Neurobiol..

[64] MacKinnon S.E., Dellon A.L., O’Brien J.P. (1991). Changes in Nerve Fiber Numbers Distal to a Nerve Repair in the Rat Sciatic Nerve Model. Muscle Nerve.

[65] Wang M.L., Rivlin M., Graham J.G., Beredjiklian P.K. (2019). Peripheral Nerve Injury, Scarring, and Recovery. Connect. Tissue. Res..

[66] Fu T., Jiang L., Peng Y., Li Z., Liu S., Lu J., Zhang F., Zhang J. (2020). Electrical Muscle Stimulation Accelerates Functional Recovery after Nerve Injury. Neuroscience.

[67] Chu X.-L., Song X.-Z., Li Q., Li Y.-R., He F., Gu X.-S., Ming D. (2022). Basic Mechanisms of Peripheral Nerve Injury and Treatment via Electrical Stimulation. Neural Regen. Res..

[68] Liu M., Zhang D., Shao C., Liu J., Ding F., Gu X. (2007). Expression Pattern of Myostatin in Gastrocnemius Muscle of Rats after Sciatic Nerve Crush Injury. Muscle Nerve.

[69] Angelov D.N., Ceynowa M., Guntinas-Lichius O., Streppel M., Grosheva M., Kiryakova S.I., Skouras E., Maegele M., Irintchev A., Neiss W.F. (2007). Mechanical Stimulation of Paralyzed Vibrissal Muscles Following Facial Nerve Injury in Adult Rat Promotes Full Recovery of Whisking. Neurobiol. Dis..

[70] Pinto M.M.R., dos Santos D.R., de Barros Bentes L.G., Lemos R.S., de Almeida N.R.C., Fernandes M.R.N., Braga J.P., Somensi D.N., Barros R.S.M. (2022). Anatomical Description of the Extratemporal Facial Nerve under High-Definition System: A Microsurgical Study in Rats. Acta Cir. Bras..

[71] DeLeonibus A., Rezaei M., Fahradyan V., Silver J., Rampazzo A., Bassiri Gharb B. (2021). A meta-analysis of Functional Outcomes in Rat Sciatic Nerve Injury Models. Microsurgery.

[72] Dinh P., Hazel A., Palispis W., Suryadevara S., Gupta R. (2009). Functional Assessment after Sciatic Nerve Injury in a Rat Model. Microsurgery.

[73] Yian C.H., Paniello R.C., Gershon Spector J. (2001). Inhibition of Motor Nerve Regeneration in a Rabbit Facial Nerve Model. Laryngoscope.

[74] Liu H., Huang H., Bi W., Tan X., Li R., Wen W., Song W., Zhang Y., Zhang F., Hu M. (2018). Effect of Chitosan Combined with Hyaluronate on Promoting the Recovery of Postoperative Facial Nerve Regeneration and Function in Rabbits. Exp. Ther. Med..

[75] de Bueno C.R.S., Pereira M., Favaretto-Júnior I.A., Buchaim R.L., Andreo J.C., Rodrigues A.d.C., Rosa-Júnior G.M. (2017). Comparative Study between Standard and Inside-out Vein Graft Techniques on Sciatic Nerve Repair of Rats. Muscular and Functional Analysis. Acta Cir. Bras..

[76] Manthou M.E., Gencheva D., Sinis N., Rink S., Papamitsou T., Abdulla D., Bendella H., Sarikcioglu L., Angelov D.N. (2021). Facial Nerve Repair by Muscle-Vein Conduit in Rats: Functional Recovery and Muscle Reinnervation. Tissue Eng. Part A.

[77] Viterbo F., Brock R.S., Maciel F., Ayestaray B., Garbino J.A., Rodrigues C.P. (2017). End-to-Side versus End-to-End Neurorrhaphy at the Peroneal Nerve in Rats. Acta Cir. Bras..

[78] Sulaiman O.A.R., Gordon T. (2019). A Rat Study of the Use of End-to-Side Peripheral Nerve Repair as a “Babysitting” Technique to Reduce the Deleterious Effect of Chronic Denervation. J. Neurosurg..

[79] Kouyoumdjian J., Graç C., Ferreira V.M. (2017). Peripheral Nerve Injuries: A Retrospective Survey of 1124 Cases. Neurol. India.

[80] Kouyoumdjian J.A. (2006). Peripheral Nerve Injuries: A Retrospective Survey of 456 Cases. Muscle Nerve.

[81] Ronchi G., Cillino M., Gambarotta G., Fornasari B.E., Raimondo S., Pugliese P., Tos P., Cordova A., Moschella F., Geuna S. (2017). Irreversible Changes Occurring in Long-Term Denervated Schwann Cells Affect Delayed Nerve Repair. J. Neurosurg..

[82] Jessen K.R., Mirsky R. (2019). The Success and Failure of the Schwann Cell Response to Nerve Injury. Front. Cell. Neurosci..

[83] Buchaim D.V., de Rodrigues A.C., Buchaim R.L., Barraviera B., Junior R.S.F., Junior G.M.R., de Souza Bueno C.R., Roque D.D., Dias D.V., Dare L.R. (2016). The New Heterologous Fibrin Sealant in Combination with Low-Level Laser Therapy (LLLT) in the Repair of the Buccal Branch of the Facial Nerve. Lasers Med. Sci..

[84] Tibúrcio F.C., Muller K.S., Leite A.P.S., de Oliveira I.R.A., Barraviera B., Ferreira R.S., Padovani C.R., Pinto C.G., Matheus S.M.M. (2023). Neuroregeneration and immune response after neurorrhaphy are improved with the use of heterologous fibrin biopolymer in addition to suture repair alone. Muscle Nerve.

[85] Pinto C.G., Leite A.P.S., Sartori A.A., Tibúrcio F.C., Barraviera B., Junior R.S.F., Filadelpho A.L., de Carvalho S.C., Matheus S.M.M. (2021). Heterologous fibrin biopolymer associated to a single suture stitch enables the return of neuromuscular junction to its mature pattern after peripheral nerve injury. Injury.

[86] Abbade L.P.F., Barraviera S.R.C.S., Silvares M.R.C., de Lima A.B.B.C.O., Haddad G.R., Gatti M.A.N., Medolago N.B., Rigotto Carneiro M.T., dos Santos L.D., Ferreira R.S. (2021). Treatment of Chronic Venous Ulcers With Heterologous Fibrin Sealant: A Phase I/II Clinical Trial. Front. Immunol..

[87] Daré L.R., Dias D.V., Rosa Junior G.M., Bueno C.R.S., Buchaim R.L., Rodrigues A.d.C., Andreo J.C. (2015). Effect of β-Hydroxy-β-Methylbutyrate in Masticatory Muscles of Rats. J. Anat..

